# P-1217. Validation of Cefiderocol (FDC) Package Insert Dosing Recommendation for Patients receiving Continuous Renal Replacement Therapy (CRRT): A Prospective Multi-Center Pharmacokinetic Study

**DOI:** 10.1093/ofid/ofae631.1399

**Published:** 2025-01-29

**Authors:** Aliaa Fouad, Emir Kobic, Nelson P Nicolasora, Melissa L Thompson Bastin, Paul M Adams, Yuwei Shen, Andrew J Fratoni, Xiaoyi Ye, Joseph L Kuti, David P Nicolau, Tomefa E Asempa

**Affiliations:** Hartford Hospital, Farmington, Connecticut; Banner University Medical Center Phoenix, Phoenix, Arizona; Banner University Medical Center, Phoenix, Arizona; University of Kentucky Medical Center, Lexington, Kentucky; University of Kentucky College of Medicine, Lexington, Kentucky; Hartford Hospital, Farmington, Connecticut; Hartford Hospital, Farmington, Connecticut; Hartford Hospital, Farmington, Connecticut; Hartford Hospital, Farmington, Connecticut; Hartford Hospital, Farmington, Connecticut; Hartford Hospital, Farmington, Connecticut

## Abstract

**Background:**

FDC is the first antibiotic with effluent flow rate-based dosing recommendations outlined in the product label for patients receiving CRRT including modalities such as continuous venovenous hemodiafiltration (CVVHDF). The objective of this study was to investigate the population pharmacokinetics of FDC among patients receiving CRRT and validate these novel dosing recommendations.

**Figure:**
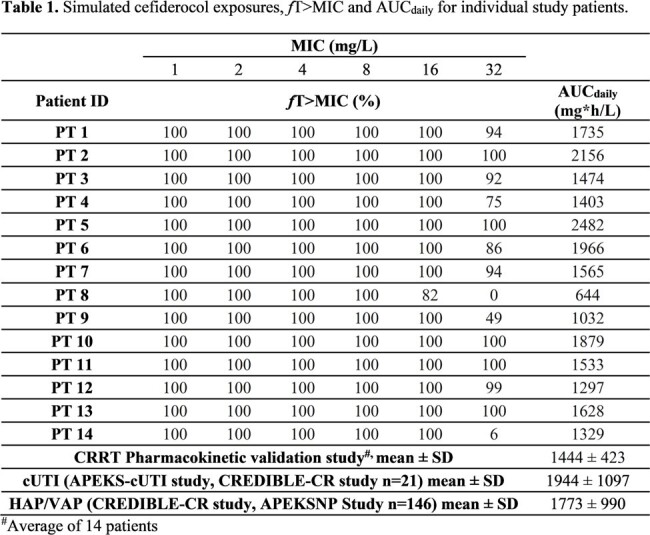

**Methods:**

A multicenter pharmacokinetic study among critically ill patients receiving CRRT was conducted. Subsequent blood sampling was performed at steady-state, and FDC concentrations were assayed by a validated Liquid Chromatography with tandem mass spectrometry. Population pharmacokinetic analyses were conducted in Pmetrics using R. The free time above the minimum inhibitory concentration (*f*T>MIC) was calculated with a target of ≥75% *f*T >MIC associated with clinical success. Total daily area under the concentration time curve (AUC_daily_) was used as a surrogate for safety and tolerability.

**Results:**

Fourteen patients on CVVHDF renal support were enrolled from August 2022 until December 2023; Hartford Hospital (n=7), Banner University Medical Center (n=5), and University of Kentucky Medical Center (n=2). Average age was 55 years and majority were male (n=9). Mean body weight was 113.5 kg, and effluent flow rates ranged from 2.1 to 5.1 L/h. FDC concentrations best fitted a two-compartment model. Mean ± SD parameter estimates for clearance, volume of the central compartment, and intercompartment transfer constants (K12 and K21) were 3.5 ± 1.5 L/h, 10.7 ± 8.4 L, 3.9 ± 1.8 h^-1^, and 2.2 ± 2.2 h^-1^, respectively. With simulations based on product label dosing recommendations, all patients achieved 100% *f*T >MIC up to MIC 8 mg/L with an AUC_daily_ (mean ± SD) of 1444 ± 423 mg*h/ L (**Table 1**). FDC was well tolerated with no serious adverse events reported among these critically ill patients.

**Conclusion:**

Based on these data, the current package insert dosing recommendations resulted in pharmacodynamically optimized FDC exposures. The simulated concentrations exceeded relevant MIC breakpoints in all patients at each effluent flow rate, and AUC_daily_ were within the range observed in patients not receiving CRRT in the Phase 3 clinical trials, suggestive of a safe and therapeutic profile.

**Disclosures:**

**Emir Kobic, BCIDP**, Shionogi: Grant/Research Support|Shionogi: Speaker Bureau **Melissa L. Thompson Bastin, PharmD, PhD**, Baxter Healthcare: Advisor/Consultant|Baxter Healthcare: Board Member|Fresenius: Advisor/Consultant **Andrew J. Fratoni, PharmD**, InsightRX: Grant/Research Support **Xiaoyi Ye, MD**, Sanofi: Advisor/Consultant **Joseph L. Kuti, PharmD**, Abbvie: Advisor/Consultant|bioMerieux: Grant/Research Support|Merck: Grant/Research Support|Pfizer: Grant/Research Support|Shionogi Inc: Advisor/Consultant|Shionogi Inc: Grant/Research Support|Shionogi Inc: Honoraria|Venatorx: Grant/Research Support **David P. Nicolau, PharmD**, CARB-X: Grant/Research Support|Innoviva: Grant/Research Support|Innoviva: Honoraria|Merck: Advisor/Consultant|Merck: Grant/Research Support|Merck: Honoraria|Pfizer: Advisor/Consultant|Pfizer: Grant/Research Support|Pfizer: Honoraria|Shionogi: Advisor/Consultant|Shionogi: Grant/Research Support|Shionogi: Honoraria|Venatorx: Grant/Research Support **Tomefa E. Asempa, PharmD**, FDA/CDER: Grant/Research Support|Paratek: Grant/Research Support|Shionogi: Grant/Research Support|Spero: Grant/Research Support|VenatoRx: Grant/Research Support

